# clusttraj: A Solvent-Informed Clustering Tool for Molecular Modeling

**DOI:** 10.1021/acs.jctc.5c00634

**Published:** 2025-07-03

**Authors:** Rafael Bicudo Ribeiro, Henrique Musseli Cezar

**Affiliations:** † Institute of Physics, 61755 University of São Paulo, Rua do Matão 1731, 05508-090 São Paulo, São Paulo, Brazil; ‡ Hylleraas Centre for Quantum Molecular Sciences and Department of Chemistry, 6305 University of Oslo, PO Box 1033 Blindern, 0315 Oslo, Norway

## Abstract

Clustering techniques are consolidated as a powerful strategy for analyzing the extensive data generated from molecular modeling. In particular, some tools have been developed to cluster configurations from classical simulations with a standard focus on individual units, ranging from small molecules to complex proteins. Since the standard approach includes computing the root mean square deviation (RMSD) of atomic positions, accounting for the permutation between atoms is crucial for optimizing the clustering procedure in the presence of identical molecules. To address this issue, we present the clusttraj program, a solvent-informed clustering package that fixes inflated RMSD values by finding the optimal pairing between configurations. The program combines reordering schemes with the Kabsch algorithm to minimize the RMSD of molecular configurations before running a hierarchical clustering protocol. By considering evaluation metrics, one can determine the ideal threshold in an automated fashion and compare the different linkage schemes available. The program capabilities are exemplified by considering solute–solvent systems ranging from pure water clusters to a solvated protein or a small solute in different solvents. As a result, we investigate the dependence on different parameters, such as the system size and reordering method, and also the representativeness of the cluster medoids for the characterization of optical properties. clusttraj is implemented as a Python library and can be employed to cluster generic ensembles of molecular configurations that go beyond solute–solvent systems.

## Graphical abstract

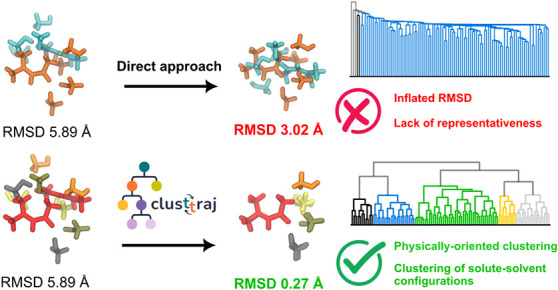

## 1 Introduction

Classical simulations of molecular systems are widely spread in several research fields,^1^ enabling the modeling from gas-phase atoms toward complex systems such as ionic liquids,^2^ macromolecules solvated in biological environments^3–5^ and various materials.^6–8^ With the development of high-performance computational packages, Molecular Dynamics (MD) and Monte Carlo (MC) methods became the main approaches at the atomistic and coarse-grained levels.^9^ From the sampled configurations, thermodynamic properties can be computed, and the analysis of the trajectories may provide a valuable understanding of mechanisms dictated by free energy variations. For instance, one can compare relative populations of conformers,^10^ track the denaturation of proteins by monitoring the radius of gyration^11^ or even establish the preferential stacking between semiconductors in thin films.^12^

Regardless of the application, the high volume of information contained in the trajectories benefits from data-driven analysis techniques. Notably, Machine Learning (ML) methods have been employed to extract powerful insights by clustering similar configurations.^13–15^ When grouping the configurations according to the distance between atoms, we can search for key features by comparing inter- and intracluster observations, but also select representative configurations. These configurations can be used for several applications such as extracting the molecular configuration from the full wave function without evoking the Born–Oppenheimer approximation,^16^ or for identifying phase changes in transition metal nanoclusters.^17,18^ Other important application is connected to reducing the overall cost when employing highly computationally demanding methods, e.g., in sequential quantum-mechanics/molecular-mechanics (s-QM/MM) calculations.^19,20^ By finding representative configurations, one can dramatically reduce the number of quantum chemistry calculations, making it feasible to use computationally demanding methods (e.g., multiconfigurational post-Hartree–Fock methods) that would be prohibitive if hundreds or thousands of configurations were considered.

Several methods have been proposed to cluster configurations from the Root Mean Square Deviation (RMSD) between snapshots of molecular dynamics trajectories. A traditional approach involves binary implementations of quality threshold and Daura’s algorithms to reduce the amount of RAM required to store the distance matrix.^21,22^ The former is implemented in programs such as VMD^23^ and GROMACS,^24^ while the latter can be performed via BitClust^22^ (or QTPy) and BitQT^25^ packages. Furthermore, the graph-based Density Peaks formalism and the Self-Organizing Maps (SOM)-based algorithm, respectively implemented in the RDPeaks^26^ and quicksom^14^ codes, further reduce the computational demand, allowing the analysis of longer trajectories. Another widely employed strategy is the application of the hierarchical clustering scheme.^27^ Despite the high sensitivity to outliers, the hierarchical clustering algorithms stand out when the number of clusters is unknown.^28^ A well-established implementation of the hierarchical clustering scheme is available in the TTClust^13^ program, while further developments are implemented in the MDSCAN.^15^ The latter combines the Density-Based Spatial Clustering of Applications with Noise (DBSCAN) method with the hierarchical clustering scheme in an efficient implementation to remove the dependency on the pairwise similarity matrix and reduce overall memory consumption.^21^ Moreover, the MDANCE package^29^ provides a collection of tools with implementations of both *k*-means and hierarchical clustering approaches designed to cluster configurations of biophysical systems.^30–33^

Most of these packages are developed with a focus in the solute. The use of clustering for trajectories that include the solvent remains relatively unexplored. Considering solvent molecules is challenging because of the labeling of atoms when molecular configurations are represented in the computer memory. This leads to possible problems due to the permutation between two identical molecules resulting in different RMSDs, despite the configurations being identical (see Figure 1). The high RMSD for configurations that are very similar in practice leads to artificial clusters when hierarchical clustering is performed. Attempting to tackle this problem, Frömbgen et al.^34^ incorporated a hierarchical clustering algorithm into the TRAVIS^35^ postprocessing software to cluster liquid conformations. However, the authors’ goal was not necessarily to find representative configurations, and not many options regarding the hierarchical clustering were explored.

**Figure 1:**
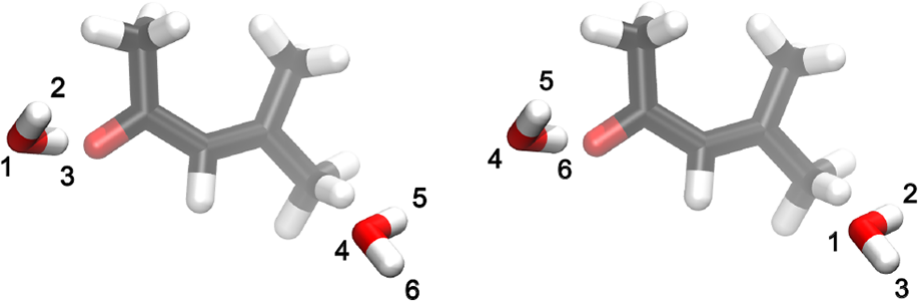
Illustration of two identical configurations with different atomic labels. The naive computation of the RMSD leads to a nonzero RMSD, despite the expected RMSD being zero in this case, since both water molecules are identical.

Here, we present clusttraj, an open source Python package that accounts for permutations to minimize the RMSD within the hierarchical clustering scheme. The package is available in the Python Package Index (PyPI), and can be installed with a simple pip install clusttraj. The program is built on top of the RMSD package by Kromann,^36^ uses efficient Python libraries such as NumPy,^37^ scikit-learn,^38^ and SciPy,^39^ and explores the embarrassingly parallel nature of the distance matrix calculation. The package is built with a focus on flexibility regarding the linkage methods within the hierarchical clustering, reordering of labels, the contribution of the solute and solvent, and which atoms are used for the RMSD computation. To illustrate some of these possibilities, we analyze the clustering of water clusters of different sizes, a small solute (mesityl oxide) with different solvents, and a larger solute (lysozyme) in water. We investigate metrics for the automatic detection of clusters, explore the different algorithms for hierarchical clustering and label reordering, and provide benchmarks of the performance.

## 2 Implementation

### 2.1 RMSD and RMSD Matrix

The implementation uses RMSD as a metric of similarity between two configurations. The RMSD between the configurations **A** = {**a**_1_, **a**_2_,..., **a**_*N*_} and **B** = {**b**_1_, **b**_2_,..., **b**_*N*_}, both with *N* atoms, is defined as

$$\mathrm{RMSD}\left(A,B\right)=\sqrt{\overset{N}{\underset{i=1}{\sum }}w_{i}\left|\right|a_{i}-b_{i}|{|}^{2}}$$ 1

where the **a**_*i*_ and **b**_*i*_ are the Cartesian coordinates for the atom of label *i* (expressed in Å), and *w*_*i*_ is the weight of the *i*-labeled atom (∑ _*i* = 1_^*N*^ *w*_*i*_ = 1). Usually, *w*_*i*_ = 1/*N* for all *i*, but assigning different weights to each atom can be useful, as we show later. As illustrated in Figure 1, the minimum value of RMSD is obtained when **A** and **B** are spatially aligned and the labels in each configuration are such that atoms of the same species in similar spatial positions correspond to the same label.

We use the package rmsd, available in PyPI, to align and reorder the labels.^36^ The package implements the spatial alignment of the configurations using the Kabsch algorithm.^40,41^ Five methods are available for the reordering of the labels: the Hungarian algorithm,^42^ the Hungarian algorithm preceded by an alignment of the principal inertia moments, FCHL19 atomic descriptors^43^ with a Hungarian cost-assignment function (also known as QML algorithm), a distance to the center of mass approach, and the brute force method. In principle, only the brute force method, which systematically considers all possible permutations of atoms of the same species, is guaranteed to find the minimum RMSD between two configurations. However, in many circumstances, especially when the configurations are properly aligned, the other algorithms can also minimize the RMSD at a significantly lower computational cost (see Sections 3.4 and S3 in the SI).

The algorithm to find the minimum RMSD of the rmsd package first reorder the labels of **B**, to then find the optimal rotation that aligns the two configurations and, finally, compute the RMSD using eq 1. The reordering is performed first to ensure the Kabsch algorithm finds the correct rotation. We call this the “direct approach”, and illustrate it in Figure 2a. However, the reordering algorithms may depend on the initial distances between atoms of the same species to find the optimal labels, as in the case of the Hungarian algorithm. Therefore, different strategies such as aligning the moments of inertia, or as we do in clusttraj, split the solute and solvent atoms, may improve the reordering of labels and lower the RMSD.

**Figure 2:**
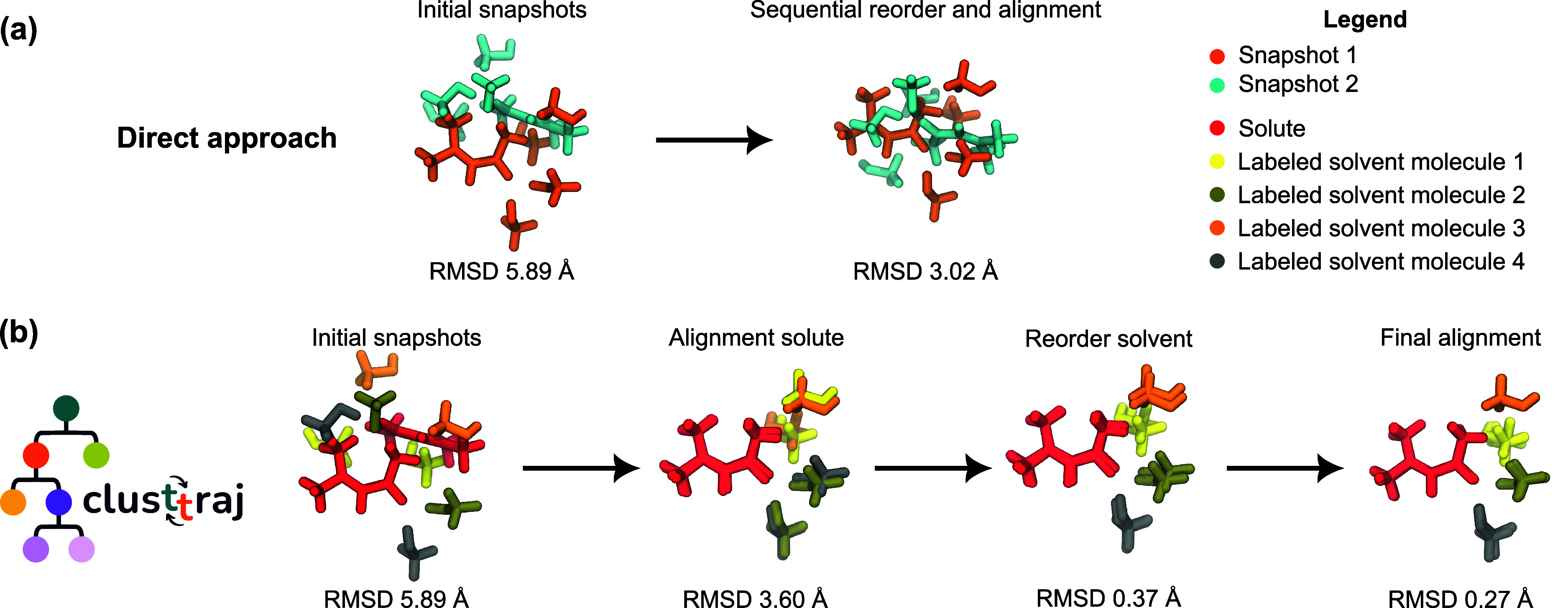
Steps illustrating the clusttraj strategy to find the minimum RMSD with the solute–solvent split. (a) RMSDs obtained with the simultaneous alignment and reorder of all the atoms, not distinguishing between solute and solvent atoms. (b) Strategy employed by clusttraj, with the RMSDs at each step.

Based on the user options, clusttraj can consider a subset or all atoms in a configuration. There are options to exclude all H atoms from the clustering or manually select exclusions. The exclusions are very flexible and can be passed to the command-line interface through ranges of atom labels in the initial trajectory. By excluding atoms, one can reduce the number of possible permutations for the reordering, and also solve the problem of having to reassign labels for the hydrogen atoms in methyl groups. These exclusions determine the size *N* of the **A** and **B** matrices.

To compute the RMSD matrix, we first parse all *M* configurations of the trajectory using OpenBabel.^44^ By using OpenBabel, many standard chemical file formats are supported for both input and output of the aligned structures, such as PDB, xyz, gro, among others. Snapshots are parsed one by one, and the entire trajectory is not loaded in memory at once. The hierarchical clustering is performed based on the RMSD between pairs of the *M* snapshots, which are combined to form the RMSD matrix. The *M*(*M* – 1)/2 RMSDs between all the configurations in the trajectory are computed in parallel using the multiprocessing Python library.

### 2.2 Solute–Solvent Split Strategy

The main difference between clusttraj and its alternatives is the solute–solvent strategy used to improve the clustering. To exploit schemes that improve the search for the minimum RMSD with relabeling and the separation of dendrograms, the *N* ^solute^ atoms of the solute are treated separately from the atoms of the solvent. This is relevant because often the solute molecule will have a greater importance than the solvent, so one can emphasize the best alignment of the solute while still accounting for solvent contributions. clusttraj uses multiple strategies to account for these contributions.

Using different weights for the solute atoms, one can give a higher or lower importance to these contributions. By default, the *w*_*i*_ in eq 1 are equal for all atoms in the configuration. In clusttraj, it is possible to define *W* ^solute^ ∈ [0, 1], the total solute weight, so

$$w_{i}=\{\begin{array}{ll} \frac{W^{\mathrm{solute}}}{N^{\mathrm{solute}}} & i\leq N^{\mathrm{solute}}\\ \frac{1-W^{\mathrm{solute}}}{N-N^{\mathrm{solute}}} & i>N^{\mathrm{solute}} \end{array}$$ 2

where we assume that the first *N* ^solute^ indexes correspond to the solute atoms.

The alignment and reordering are split in different steps for the solute and solvent, as shown in Figure 2b. This strategy takes advantage of a smaller search space for the label reordering and favors a better clustering with respect to the solute. Also, since the reordering algorithms may depend on the Euclidean distance between the atoms of the same species, we first center the coordinates at the solute and perform a Kabsch alignment of the solute atoms only. This often leads to good alignment even before reordering, especially when H atoms are not considered, and it anyway serves as an approximate rotation to improve the initial RMSD. From this initial rotation, to handle cases where the order of the solute atoms has changed, the reassignment of the solute labels is performed. Based on this relabeling, a new Kabsch rotation guarantees optimal alignment between the two structures.

After these operations have been performed for the solute, we focus on the solvent molecules. Excluding the solute atoms from the assignment problem, we relabel the atoms of the solvent molecules. At this point, the configurations should be optimally aligned between the solutes, as they are in Figure 1, and the relabeling of the solvent molecules is performed on a smaller subset of atoms and Cartesian coordinates that are similar for molecules in the same spatial region. Under these circumstances, the reassignment algorithms can perform their best and possibly find the minimum RMSD. Therefore, even algorithms that rely on some heuristics for relabeling, the case of the Hungarian or distance algorithms implemented in the rmsd package and used in clusttraj, usually work better with the reduced number of atoms in each step and after alignment. A final Kabsch rotation including the solvent atoms can then optionally be performed before the final RMSD is returned.

A comparison between the direct approach, as implemented in the rmsd^36^ package, where the solute and the solvent are treated simultaneously without distinction, and the clusttraj approach is shown in Figure 2. In this example, the RMSD found by the direct approach (3.02 Å) is much higher than that found by clusttraj’s solute–solvent informed strategy (0.27 Å) when using *w*_*i*_ = 1/*N*. Other strategies such as initial alignment of the principal inertia moments, implemented in the rmsd package, lower the RMSD in the direct approach (2.65 Å), yet fail to obtain the minimum value found with the solute–solvent split strategy.

### 2.3 Agglomerative Hierarchical Clustering

In agglomerative hierarchical clustering a tree-like structure of nested clusters is built by iteratively merging data points based on similarity up until all clusters are merged into one. Initially, each structure corresponds to its own cluster, and, based on a linkage algorithm, pairs of clusters are joined hierarchically.^27^ The structure formed by this linkage is known as dendrogram, and gives a visual and quantitative representation of the distance between clusters. Based on a cut at a certain tree height, the number of clusters and the members of each cluster are determined.

In clusttraj, the hierarchical clustering is performed using SciPy.^39^ SciPy has many different algorithms implemented for linkage, such as single, average, complete, ward, among others.^45^ The clusters and separation between clusters are heavily determined by which algorithm is used. clusttraj defaults to the ward linkage, which as we show in the results section, provides a good separation between clusters.

The threshold distance of the dendrogram is also another important quantity that determines the quality of the clustering. If a too high threshold is used, the algorithm returns only very few clusters, while a too small threshold gives basically no clustering at all. Some metrics can be used to optimize this threshold, such as the silhouette score (SS),^46^ the Calinski Harabasz score (CH),^47^ the Davies-Bouldin score (DB)^48^ and the cophenetic correlation coefficient (CPCC).^49^ In principle, one should aim to maximize the SS, which is a coefficient that ranges from −1 to 1, with positive values indicating that, on average, each point is closer to other points in the same cluster than to those in different clusters. CH and DB are built around the cluster centroids, favoring spherical distributions, while CPCC consider heights from the hierarchical tree to assess the dissimilarities between the dendrogram and the original data. These metrics are useful in helping determine the threshold, but a visual inspection of the similarity between cluster members is always necessary. clusttraj can determine the threshold automatically based on the SS, or a manual threshold can be used.

### 2.4 Output Information

After classification, clusttraj outputs information about the clustering process and the clusters. The RMSD matrix is stored to save computational time when rerunning analysis with a different threshold or linkage algorithm, but also to allow analysis regarding the minimum RMSD distance between cluster members. In addition to the RMSD matrix, the classification of each snapshot, the dendrogram, reduced-dimensionality plots, and classification evolution over time are saved.

For a visual analysis of the similarity of structures between clusters, clusttraj also outputs one trajectory file for each cluster, containing the best superposition of each cluster member with the medoid. We define the medoid as the structure that has the smallest sum of RMSD to all other cluster members, and consider it the representative structure of the cluster for analysis in the results section.

## 3 Example Applications

### 3.1 Lysozyme Protein

Since clustering methods are often applied in the context of bioinformatics, we investigated the lysozyme protein conformations in solution. An *NVT* thermalization of the 1AKI crystal structure lysozyme protein solvated in water and neutralized with NaCl at a concentration of 0.15 mol L^–1^ was performed for 10 ns, employing the leapfrog integrator with a time step of 2 fs. Hydrogen bonds were constrained and the temperature was set at 300 K using the modified Berendsen thermostat^50^ with a dumping constant of 0.1 ps. The OPLS-AA^51,52^ and TIP3P^53^ force fields were employed for the protein and water, respectively. From the trajectory file, 100 snapshots were extracted to apply the clustering procedure. The simulation was performed with GROMACS^24^ and the GROMACS toolkit was combined with the functionalities from MDAnalysis^54^ package to modify trajectory files and analyze the clustering results.

At first, we investigated the solvent distribution around the amino acids by including the 100 water molecules whose atoms were closest to any protein atom, as illustrated by Figure 3. We combine the Hungarian algorithm for label reordering with the Ward variance minimization method to calculate the distance between clusters. The RMSD threshold was chosen to maximize the SS, and the hydrogen atoms were not included, resulting in two clusters with 25 and 75 samples each. Since these snapshots were extracted from a trajectory file, we can track the time evolution of the solvated protein conformation. The first 25 configurations belong to cluster 1, and the remaining belong to cluster 2. Considering that the RMSD of atomic positions is provided as input for the clustering procedure, one should expect changes in the structural properties of the snapshots from different clusters. In particular, as shown in Figure 4, the cluster shift is related to the radius of gyration of the protein. From the top panel, we can observe higher values for Cluster 1 and a difference of 0.019 nm between each cluster’s average radius of gyration, corresponding to a substantial 29.7% of the observed range of 0.064 nm. Therefore, the clustering procedure allowed the configurations to be grouped according to the protein packing while still considering the interaction with the solvent.

**Figure 3:**
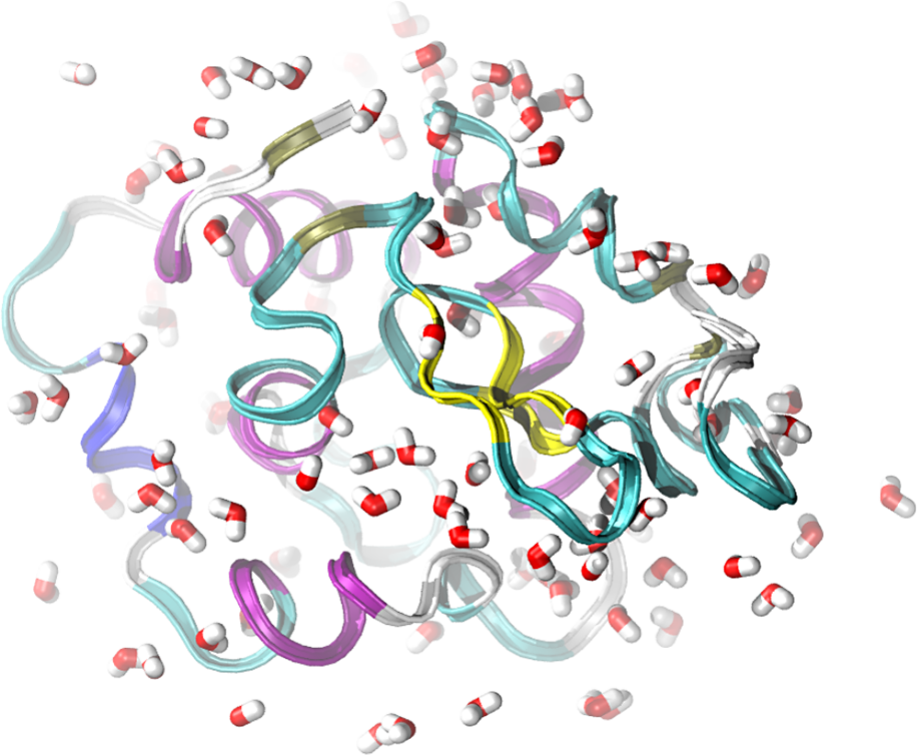
Snapshot of the first frame of the lysozyme trajectory. The protein is solvated by 100 water molecules.

**Figure 4:**
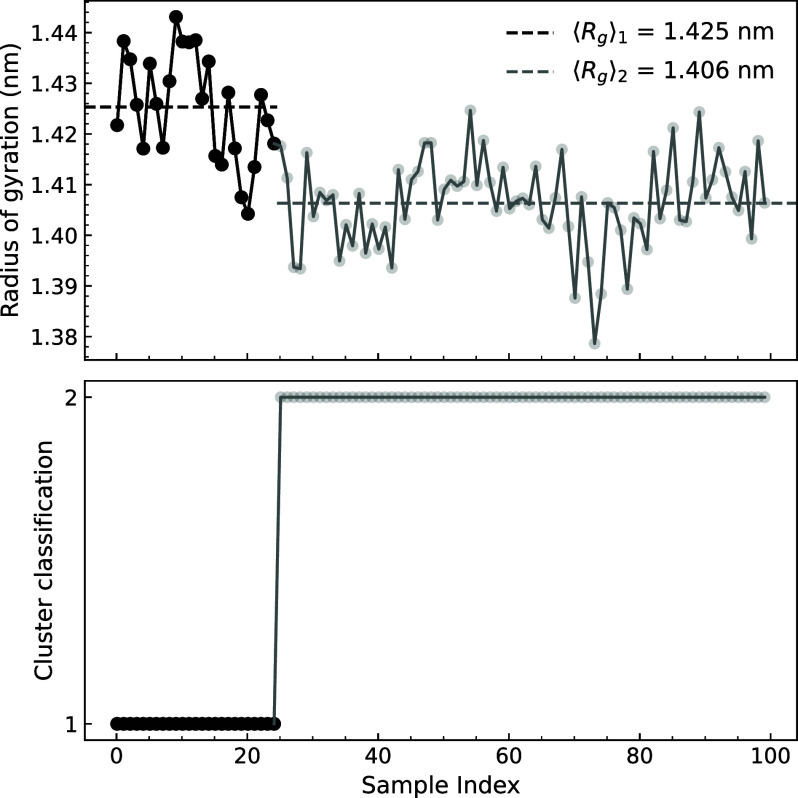
Radius of gyration (top) compared with the cluster evolution (bottom) for the 100 configurations of the lysozyme protein solvated by 100 water molecules.

One can also focus on different properties by changing the atoms in the configuration file. For example, by considering only the lysine amino acid with the closest water molecules, we could identify differences in the number of hydrogen bonds of configurations from different clusters. See the Supporting Information (SI) for more details.

### 3.2 Water

For the second example, we performed a molecular dynamics simulation of 500 water molecules in the *NVT* ensemble, with a density of 1 g/cm^3^. The equations of motion were integrated using the leapfrog algorithm with a time step of 0.1 fs. The temperature was kept constant at 300 K using the modified Berendsen thermostat^50^ and a damping constant of 0.1 ps. We used the TIP3P force field to describe the interactions^53^ and the simulation was performed using GROMACS^24^ software. From the 1 ns simulation, we extracted 100 snapshots and a random water molecule (the solute) was chosen as a reference to generate the input file for the clustering procedure. We varied the number of nearest neighbor molecules (solvent molecules) from 1 to 100 to investigate the system size dependence. The Hungarian algorithm and Ward variance minimization method were employed to reorder the atoms and to compute the distance between clusters within the linkage scheme, respectively. The threshold RMSD was chosen to maximize the silhouette score, yielding the results presented in Table 1.

**Table 1:** Clustering Results for Different Number of Neighbor Water Molecules

# of water molecules	# of clusters	RMSD (Å)	silhouette score	RMSD matrix sum (Å)
1 + 1	3	1.822	0.413	1720
1 + 2	8	1.670	0.261	3177
1 + 3	4	4.031	0.261	4547
1 + 4	41	1.290	0.153	4404
1 + 5	32	1.818	0.148	4843
1 + 6	45	1.704	0.132	5057
1 + 7	47	1.814	0.114	5133
1 + 8	39	1.934	0.106	5102
1 + 9	45	1.873	0.095	5103
1 + 10	38	1.928	0.073	5080
1 + 20	50	1.877	0.040	4987
1 + 30	46	1.905	0.028	4948
1 + 40	41	1.949	0.024	4921
1 + 50	38	1.944	0.027	4927
1 + 100	2	2.536	0.015	4758

Although the RMSD threshold can be chosen by hand, one should consider the evaluation metrics to improve the clustering procedure. Despite having positive SS values for all the systems presented in Table 1, there is an explicit dependency on the system size. The clustering quality decreases as we add more atoms, reflecting the broader variety of configurations due to increasing the system’s degrees of freedom. This conformational diversity is connected to the RMSD between snapshots. Since low RMSD indicates a higher similarity between configurations, the sum of RMSD between all snapshots can be considered to assess the quality of the reordering and minimization procedures. As shown in the last column of Table 1, the sum of RMSDs grows as we increase the number of solvent waters but eventually converges.

Despite the high correlation between SS and the sum of RMSDs (Pearson’s correlation coefficient of −0.847 for values in Table 1), the assessment of cluster quality should not be the only metric. For instance, when considering 1 + 7 and 1 + 100 systems, the SS indicates a better clustering for the smaller system while the RMSD sum favors the larger one. This contrast can also be observed for the clustering of 1 + 2 and 1 + 3 systems, which share the same SS but have significantly different RMSD sums of 3177 and 4547 Å, respectively. Therefore, the clustering quality does not necessarily reflect the quality of the reordering and minimization procedures.

Examining the RMSD thresholds can also provide valuable information regarding the inter- and intracluster similarity between configurations. For 1 + 2 and 1 + 3 systems, SS was maximized using 1.670 and 4.031 Å thresholds, respectively. This distinction indicates that configurations belonging to the same cluster are more diverse for the larger system, suggesting that the medoid is less representative of the overall cluster. On the other hand, having more clusters may fail to reduce the complexity, requiring a balanced description between the number of clusters and the evaluation metrics. This highlights the importance of fine-tuning the RMSD threshold to perform physically accurate clustering.

### 3.3 Mesityl Oxide

For the final example, we studied the mesityl oxide (MOx) [(CH_3_)_2_C=CHC­(=O)­CH_3_, 4-methyl-3-penten-2-one] molecule solvated in methanol and in acetonitrile. The solute and the four closest solvent molecules were explicitly considered, and the configurations were extracted from Configurational Bias Monte Carlo (CBMC) simulations performed with DICE.^55^ Simulations were performed in the *NPT* ensemble with *P* = 1 atm and *T* = 300 K, with one MOx molecule and 800 solvent molecules. The internal degrees of freedom of the solute were sampled with the CBMC algorithm, while the solvent molecules are kept rigid. More details of the simulation conditions are given in a previous work.^56^

#### 3.3.1 Methanol

Considering a threshold of 10 Å, we obtained 3 clusters with significant structural differences between the configurations, as shown in SI. However, more interesting results were obtained when changing the atom weights for solute and solvent molecules. Specifically, by excluding the H atoms and increasing the weight of solute atoms to 90% of the total RMSD, we obtained two clusters that separate *syn* and *anti* conformations, as shown in Figure 5.

**Figure 5:**
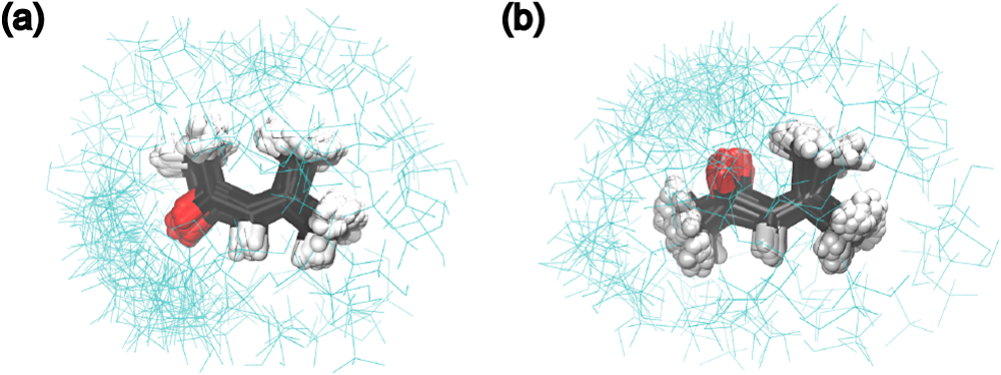
Superposition of MOx configurations separated in two clusters with (a) *anti* and (b) *syn* conformers. Methanol molecules are shown as cyan lines for better visualization.

Shao et al.^28^ investigated how different algorithms behave for clustering solute configurations obtained from molecular dynamics trajectories and found that the average linkage algorithm (also known as UPGMA) performed better than its alternatives. To investigate the clustering sensitivity toward the linkage method with the solute–solvent splitting of clusttraj, we compared the different approaches available in SciPy to generate a fixed number of 5 clusters and the results are shown in Table 2. We evaluated all clustering metrics implemented in clusttraj, namely, the SS, CH, DB, and CPCC. In practice, our goal is to maximize SS, CH, and CPCC while minimizing DB, which is not trivial according to Table 2. Since the CH score considers the ratio between cluster separation and dispersion of configurations within the same cluster, the maximization of this coefficient agrees with the visual analysis of the dendrogram, disfavoring the use of simple, centroid and median distances (see SI). In this sense, our results are consistent with the literature,^28^ as average and ward distance methods are commonly used for hierarchical clustering of single molecule configurations from MD trajectories.

**Table 2:** Evaluation Metrics of Hierarchical Clustering with Different Distance Methods^a^

method	RMSD (Å)	SS	CH	DB	CPCC
single	1.98	–0.068	1.191	1.223	0.281
complete	4.40	0.056	8.555	2.828	0.371
average	3.10	0.081	6.285	1.739	0.544
weighted	3.10	0.084	7.107	2.220	0.469
centroid	2.43	0.060	3.186	1.291	0.431
median	2.40	–0.002	3.043	1.305	0.452
ward	6.50	0.107	10.110	2.087	0.419

a Root-mean-square deviation (RMSD), silhouette score (SS), Calinski Harabasz score (CH), Davies–Bouldin score (DB), and Cophenetic correlation coefficient (CPCC).

The usefulness of clustering configurations is also extendable to investigating quantum properties. In this case, we are interested in the solvatochromic shift of the absorption spectrum. The calculations reported in a previous work^10^ show a shift in the convoluted absorption spectrum when methanol molecules are included in the quantum region. Within the s-QM/MM method, an ensemble of configurations is extracted from uncorrelated snapshots of classical simulations to perform the quantum calculations. The number of configurations required to achieve convergence varies according to the property. Typically, at least 100 snapshots are required to properly access the configurational space, resulting in a computational demand depending on the level of calculations. In this context, the benefit of clustering becomes evident when we improve the intercluster configuration variety by reducing the RMSD threshold to obtain 4 clusters of MOx and methanol molecules. Considering the medoid configurations from each cluster, we obtain an average excitation energy of 243.75 nm. Given the estimated error of σ = 100 cm^–1^ (0.591 nm for this wavelength), this result is compatible within a 1.3 σ interval with the actual value of 242.98 nm determined from the 100 configurations. However, if we randomly select, for instance, 10 sets with 4 configurations in each, we obtain an average excitation energy of 241.44 nm for the brightest excited state, deviating by 2.6 σ from the convoluted value. Considering the normal distribution, a 1.6 σ deviation has a probability of approximately 19.7%, meaning it is relatively common and could easily occur due to random variation. In contrast, a 2.6 σ deviation has a probability of only 0.95%, making it much rarer and more statistically significant. Therefore, in contrast to the random sampling of configurations, the medoid approach provides a consistent solvatochromic shift of the brightest absorption peak and drastically reduces the computational cost. By only considering 4 configurations instead of 100, we can also afford a higher computational cost per calculation, producing more reliable results, e.g., by expanding the quantum region or refining the level of theory employed.

#### 3.3.2 Acetonitrile

Similarly to the study in methanol, we performed the clustering of 100 configurations comprising a single MOx and 4 acetonitrile molecules. The clustering scheme included all hydrogen atoms, and we fixed an RMSD threshold of 4.0 Å. The Hungarian reorder algorithm was employed with *W* ^solute^ = 0.9, resulting in 4 clusters with populations 22, 39, 22 and 17, respectively.

As an extension of the previous example, we compared the UV–vis absorption spectra of the medoid configurations against their respective average cluster spectrum. The spectrum was determined by fitting Gaussian functions to the first 6 singlet excited states, resulting in the curves shown in Figure 6.

**Figure 6:**
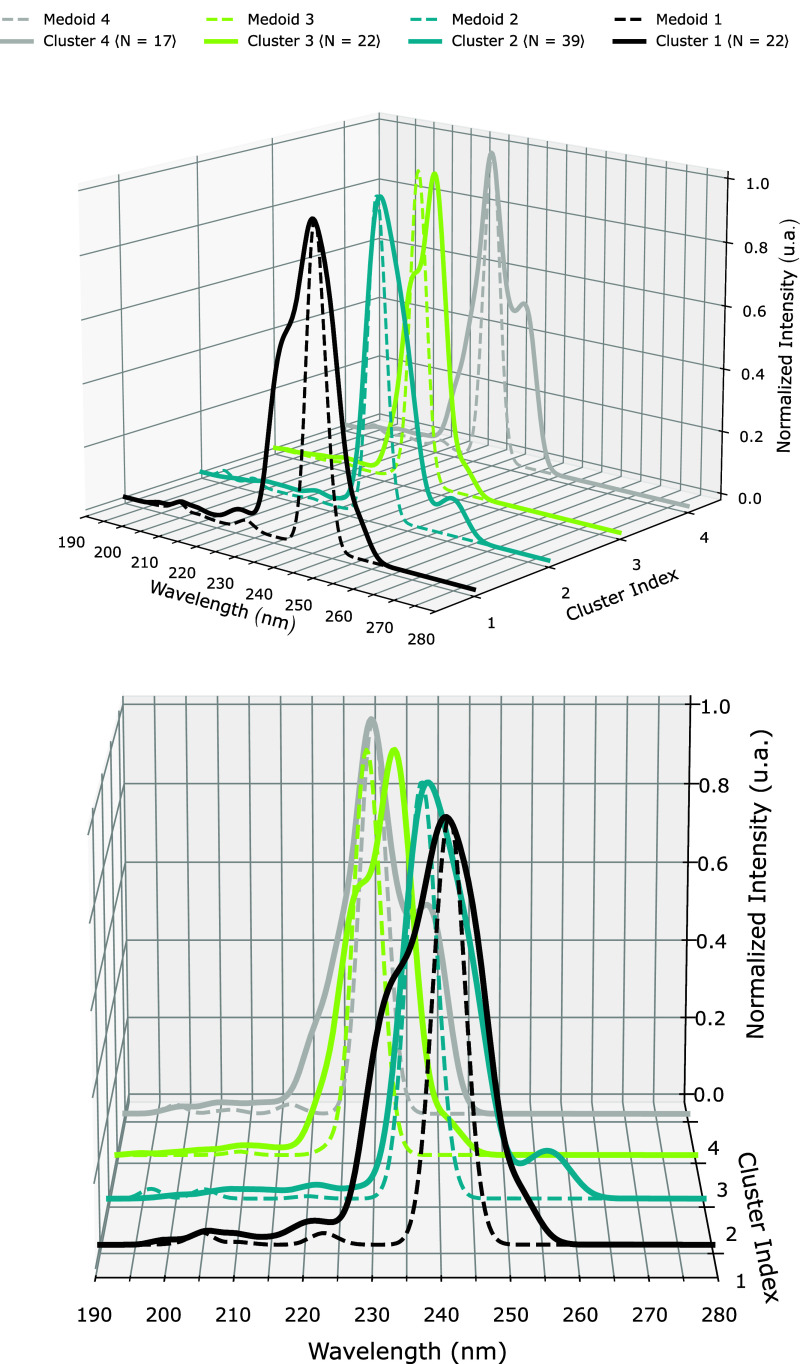
UV–vis absorption spectra of MOx and the 4 closest acetonitrile molecules. Dotted lines are the medoids’ spectra only, and solid lines correspond to the averaged spectrum over all 22, 39, 22, and 17 configurations from clusters 1, 2, 3, and 4, respectively.

Despite the enlargement in the peak width that arises from the different configurations, the medoid spectra (dotted lines) generally matched the brightest excitation energy. For clusters 1, 2 and 4, the medoid peak matches the brightest peak, while for cluster 3, it corresponds to the second brightest peak. Since the differences in excitation energies and oscillator strengths come from the geometry changes between configurations, the medoid bypasses detailed shapes, such as the shoulder-like structure observed around 230 nm for the averaged spectrum of Cluster 1. Additional information concerning the solvent contribution to the desired excitation can be incorporated into the scheme by changing the weight of solute atoms.

This example is also important to illustrate the role of the RMSD threshold in the clustering procedure. Despite the use of metrics such as the SS, CH, DB, and CPCC to evaluate clustering quality and even provide automatic thresholds for the RMSD, user assessment of the clustering remains extremely important. While decreasing the RMSD threshold increases the number of clusters, it tends to reduce the variance between configurations in the same clusters, improving the representativeness of the medoid configuration. The spectra in Figure 6 show that each medoid contributes with different excitations, exhibiting maximum absorption at distinct wavelengths. Consequently, the quality of the overall spectra obtained from the combination of the individual representatives depends on the RMSD threshold. Few clusters will tend to have poor representative structures, while having many clusters will fail the purpose of performing the clustering. The appropriate balance can only be determined by the user, as it is both system-dependent and application-dependent.

### 3.4 Computational Cost

As described in Section 2, the program is developed on top of well-optimized Python libraries such as NumPy and scikit-learn. However, the additional steps required to reorder and permute identical molecules can be very costly, making the performance comparison with other clustering packages not meaningful.

Regardless of the options used for the analysis, one must compute the RMSD matrix for all clustering schemes considering the RMSD of atomic positions. For a trajectory with *M* configurations, there are *M*(*M* – 1)/2 pairwise RMSDs, resulting in a computational complexity scaling of $O\left(M^{2}\right)$ . To find the optimal pairing between two configurations, we employ the Kabsch algorithm^40,41^ that generally scales linearly with the number of atoms, *N*.^57^ However, the most computationally demanding part relates to the reordering step. In a simplified picture, considering all possible atomic permutations between atoms has a drastic scaling of O(N!) . Although a natural strategy, this brute-force approach is unfeasible for larger systems, requiring other techniques such as the distance or Hungarian algorithms. The former reorders the atoms concerning the distance to the system centroid, yielding an approximate solution with a computational complexity of O(NlogN) .^58^ On the other hand, the latter describes the reordering process in terms of a linear assignment problem and finds the exact solution that typically scales with $O\left(N^{3}\right)$ .^59,60^

To investigate the clusttraj performance, we compared the computational demand for each reordering method with different system sizes. We considered the same trajectory files of the first example, comprising a water molecule solvated by 1, 3, 10, or 100 water molecules. All calculations used 4 threads in an Apple M2 processor. Results are shown in Table 3.

**Table 3:** Time Consumption of Different Reordering Algorithms for Clustering 1 Water Solvated by 1, 3, 10, 20, or 100 Water Molecules^a^

reordering algorithm	system	RMSD matrix (s)	total time (s)
Hungarian	1 + 1	2.17(0.28)	4.14(0.42)
	1 + 10	2.23(0.19)	3.95(0.19)
	1 + 100	16.17(0.67)	18.29(0.72)
Brute force	1 + 1	2.34(0.17)	4.15(0.16)
	1 + 3	38.14(0.87)	40.13(0.87)
Distance	1 + 1	2.05(0.20)	3.79(0.25)
	1 + 10	2.27(0.15)	3.99(0.19)
	1 + 100	7.30(1.20)	9.71(1.53)
QML	1 + 1	2.50(0.16)	4.29(0.21)
	1 + 10	16.34(0.27)	18.17(0.32)
	1 + 20	118.11(8.04)	119.97(8.17)

a The results were averaged over 10 independent simulations.

In all cases, the most expensive part concerns the RMSD matrix calculation, which includes the reordering scheme and the Kabsch algorithm. As expected, for practical purposes, the factorial scaling of the brute force algorithm limits the procedure to systems with up to dozens of atoms shared among identical molecules. On the other hand, the distance approach is the fastest one, allowing the treatment of larger systems and including more configurations. However, this approximated method yields worse metrics than the others, changing the number of observations per cluster and even the optimal number of clusters. For example, when considering the 1 + 100 system, the distance method produces two clusters with 42 and 58 observations, maximizing the SS to 0.052 with a RMSD matrix sum of 50875 Å. Considering the same set of configurations, the Hungarian algorithm produces 10 clusters with an SS of 0.205 and a RMSD matrix sum of 31132 Å. These differences between the distance and Hungarian algorithms are observed for all the other evaluation metrics and for the three systems considered in Table 3. See Sections S3 and S4 of the SI for a detailed comparison between the quality of each reordering algorithm and a illustrative comparison with a standard clustering package, respectively.

## 4 Conclusions

We presented a novel tool for clustering molecular configurations within the hierarchical clustering approach. Given the importance of considering the permutation of identical molecules to improve cluster formation, we developed the clusttraj program, an open-source tool that allows the clustering of a new range of systems within a simple command line interface.

Following the standard approach, molecular configurations are clustered by considering the RMSD between atomic positions. However, our procedure includes an initial reordering scheme to find the optimal pairing, avoiding the spurious increase of the RMSD due to the permutation of identical molecules, enabling, for example, the clustering of solute–solvent systems. Different reordering algorithms are available, from the refined treatment of all possible permutations to approximate methods that enable the study of larger systems with extensive ensembles of configurations. In particular, the Hungarian algorithm presented the best cost-benefit among the methods available and was employed throughout the examples.

By considering representative systems, we consistently varied the proportion between identical and nonidentical molecules to investigate the clustering performance. We obtained higher evaluation metrics when considering the average and Ward variance minimization linkage methods for computing the intercluster distance. Although no direct relationship was observed between performance and system size, clustering of the large lysozyme system captured structural differences, yielding two clusters with different protein gyration radii. This behavior not only indicates a correlation between the clustering procedure and the structural properties but also reproduces the results expected for the analysis of isolated molecules by capturing the solute properties despite the presence of solvent.

For the smaller systems, we observed that optimizing clustering metrics does not necessarily result in minimizing RMSD between configurations. However, by adjusting the weight of the solute atoms, we successfully separated the *syn* and *anti* conformations of a MOx molecule in methanol while maximizing the SS. Furthermore, by increasing the RMSD threshold to produce 4 clusters, we obtained a good agreement between the solvatochromic shift of the UV–vis absorption spectrum convoluted over 100 configurations and determined only from the cluster medoids. A similar trend was observed when considering MOx solvated in acetonitrile. For each cluster, the medoid configuration was able to reproduce at least one of the two brightest peaks of the absorption spectrum convoluted over all configurations of the same cluster. Therefore, considering the medoid configuration is a solid method for selecting representative configurations that can improve the comparison while drastically reducing the computational cost. Since clusttraj provides one solution for clustering identical molecules, we present a valuable tool that covers traditional solution-based clustering applications and can leverage the molecular modeling of complex systems.

## Supplementary Material



## Data Availability

The clusttraj code is available free of charge on https://github.com/hmcezar/clusttraj under the GPL-3.0 license. The trajectory files and the clusttraj output of all the examples presented in the manuscript can be found in the Norwegian Research Infrastructure Services (NIRD) research data archive on 10.11582/2025.2ab1vwjm.

## Notes

Hydrogen bond analysis for clusters of lysine amino acid solvated in water; clusters of mesityl oxide (MOx) solvated in methanol considering a different RMSD threshold; dendrograms from the clustering of MOx solvated in methanol using different linkage methods; a test of the reliability of reordering algorithms; and a comparison with TTClust (PDF)

## References

[1] Mitra, S. ; Chaplot, S. L. Computational Statistical Physics; Hindustan Book Agency: Gurgaon; Chapter Applications of Molecular Dynamics Simulations, 2011; pp 199–230.

[2] Maginn E. J. (2009). Molecular
simulation of ionic liquids: current status and future opportunities. J. Phys.: Condens. Matter.

[3] Karplus M., Petsko G. A. (1980). Molecular dynamics
simulations in biology. Nature.

[4] Fu H., Chen H., Blazhynska M., de Lacam E. G. C., Szczepaniak F., Pavlova A., Shao X., Gumbart J. C., Dehez F., Roux B., Cai W., Chipot C. (2022). Accurate determination
of protein:ligand standard binding free energies from molecular dynamics
simulations. Nat. Protoc..

[5] Pedebos C., Khalid S. (2022). Simulations of the spike: molecular
dynamics and SARS-CoV-2. Nat. Rev. Microbiol..

[6] Mollahosseini A., Abdelrasoul A. (2021). Molecular dynamics simulation for
membrane separation
and porous materials: A current state of art review. Journal of Molecular Graphics and Modelling.

[7] Chen Z., Pei J., Li R., Xiao F. (2018). Performance characteristics of asphalt
materials based on molecular dynamics simulation - A review. Construction and Building Materials.

[8] Krishna S., Sreedhar I., Patel C. M. (2021). Molecular
dynamics simulation of
polyamide-based materials–A review. Comput.
Mater. Sci..

[9] Allen, M. P. ; Tildesley, D. J. Computer Simulation of Liquids, 2nd ed.; Oxford University Press, 2018.

[10] Cezar H. M., Canuto S., Coutinho K. (2018). Solvent effect on the syn/anti conformational
stability: A comparison between conformational bias Monte Carlo and
molecular dynamics methods. Int. J. Quantum
Chem..

[11] Lobanov M. Y., Bogatyreva N. S., Galzitskaya O. V. (2008). Radius of gyration as an indicator
of protein structure compactness. Mol. Biol..

[12] Armin A., Li W., Sandberg O. J., Xiao Z., Ding L., Nelson J., Neher D., Vandewal K., Shoaee S., Wang T., Ade H., Heumüller T., Brabec C., Meredith P. (2021). A History
and Perspective of Non-Fullerene Electron Acceptors for Organic Solar
Cells. Adv. Energy Mater..

[13] Tubiana T., Carvaillo J.-C., Boulard Y., Bressanelli S. (2018). TTClust: A
Versatile Molecular Simulation Trajectory ClusteringProgram with Graphical
Summaries. J. Chem. Inf. Model..

[14] Mallet V., Nilges M., Bouvier G. (2021). quicksom:
Self-Organizing Maps on
GPUs for clustering of molecular dynamics trajectories. Bioinformatics.

[15] González-Alemán R., Platero-Rochart D., Rodrłguez-Serradet A., Hernández-Rodríguez E. W., Caballero J., Leclerc F., Montero-Cabrera L. (2022). MDSCAN: RMSD-based
HDBSCAN clustering of long molecular dynamics. Bioinformatics.

[16] Lang L., Cezar H. M., Adamowicz L., Pedersen T. B. (2024). Quantum Definition
of Molecular Structure. J. Am. Chem. Soc..

[17] Cezar H. M., Rondina G. G., Da Silva J. L. F. (2017). Parallel
tempering Monte Carlo combined
with clustering Euclidean metric analysis to study the thermodynamic
stability of Lennard-Jones nanoclusters. J.
Chem. Phys..

[18] Cezar H. M., Rondina G. G., Da Silva J. L. F. (2019). Thermodynamic
properties of 55-atom
Pt-based nanoalloys: Phase changes and structural effects on the electronic
properties. J. Chem. Phys..

[19] Coutinho, K. ; Rivelino, R. ; Georg, H. C. ; Canuto, S. Solvation Effects in Molecules and Biomolecules; Springer: Dordrecht, Chapter The Sequential QM/MM Method and its Applications to Solvent Effects in Electronic and Structural Properties of Solutes; 2008; pp 159–189.

[20] Ribeiro R. B., Franco L. R., Holmes A., Ramos T. N., Wang E., Varella M. T. d. N., Araujo C. M. (2025). Assessing Structural and Optical
Properties of PTQ10-Based Donor Polymers in Solution for Eco-Friendly
Photovoltaics: A Multiscale Modeling Study. J. Phys. Chem. B.

[21] Campello R. J. G. B., Moulavi D., Sander J. (2013). Density-Based
Clustering Based on
Hierarchical Density Estimates. Advances in
Knowledge Discovery and Data Mining..

[22] González-Alemán R., Hernández-Castillo D., Rodríguez-Serradet A., Caballero J., Hernández-Rodríguez E. W., Montero-Cabrera L. (2020). BitClust: Fast Geometrical Clustering of Long Molecular
Dynamics Simulations. J. Chem. Inf. Model..

[23] Humphrey W., Dalke A., Schulten K. (1996). VMD: Visual
molecular dynamics. J. Mol. Graphics.

[24] Abraham M., Murtola T., Schulz R., Páll S., Smith J., Hess B., Lindahl E. (2015). GROMACS: High
performance
molecular simulations through multi-level parallelism from laptops
to supercomputers. SoftwareX.

[25] González-Alemán R., Platero-Rochart D., Hernández-Castillo D., Hernández-Rodríguez E. W., Caballero J., Leclerc F., Montero-Cabrera L. (2021). BitQT: a graph-based
approach to the quality threshold clustering of molecular dynamics. Bioinformatics.

[26] Platero-Rochart D., González-Alemán R., Hernández-Rodrłguez E. W., Leclerc F., Caballero J., Montero-Cabrera L. (2022). RCDPeaks:
memory-efficient density peaks clustering of long molecular dynamics. Bioinformatics.

[27] Murtagh F., Contreras P. (2012). Algorithms for hierarchical clustering: an overview. WIREs Data Mining and Knowledge Discovery.

[28] Shao J., Tanner S. W., Thompson N., Cheatham T. E. (2007). Clustering Molecular
Dynamics Trajectories: 1. Characterizing the Performance of Different
Clustering Algorithms. J. Chem. Theory Comput..

[29] Chen L., Roe D. R., Kochert M., Simmerling C., Miranda-Quintana R. A. (2024). k-Means NANI: An Improved Clustering
Algorithm for
Molecular Dynamics Simulations. J. Chem. Theory
Comput..

[30] Chen L., Smith M., Roe D. R., Miranda-Quintana R. A. (2025). Extended
Quality (eQual): Radial Threshold Clustering Based on n-ary Similarity. J. Chem. Inf. Model..

[31] Chen L., Santos J. B. W., Gaza J., Perez A., Miranda-Quintana R. A. (2025). Hierarchical
Extended Linkage Method (HELM)’s Deep Dive into Hybrid Clustering
Strategies. J. Chem. Inf. Model..

[32] Chen L., Mondal A., Perez A., Miranda-Quintana R. A. (2024). Protein
Retrieval via Integrative Molecular Ensembles (PRIME) through Extended
Similarity Indices. J. Chem. Theory Comput..

[33] Chen L., Leung J. M. G., Zsigmond K., Chong L. T., Miranda-Quintana R. A. (2025). SHINE:
Deterministic Many-to-Many Clustering of Molecular Pathways. J. Chem. Inf. Model..

[34] Frömbgen T., Blasius J., Alizadeh V., Chaumont A., Brehm M., Kirchner B. (2022). Cluster Analysis in Liquids: A Novel
Tool in TRAVIS. J. Chem. Inf. Model..

[35] Brehm M., Kirchner B. (2011). TRAVIS - A Free Analyzer and Visualizer
for Monte Carlo
and Molecular Dynamics Trajectories. J. Chem.
Inf. Model..

[36] Kromann, J. C. Calculate Root-mean-square deviation (RMSD) of Two Molecules Using Rotation, 2025. https://github.com/charnley/rmsd.

[37] Harris C. R., Millman K. J., van der
Walt S. J., Gommers R., Virtanen P., Cournapeau D., Wieser E., Taylor J., Berg S., Smith N. J., Kern R., Picus M., Hoyer S., van Kerkwijk M. H., Brett M., Haldane A., del Río J. F., Wiebe M., Peterson P., Gérard-Marchant P., Sheppard K., Reddy T., Weckesser W., Abbasi H., Gohlke C., Oliphant T. E. (2020). Array programming
with NumPy. Nature.

[38] Pedregosa F., Varoquaux G., Gramfort A., Michel V., Thirion B., Grisel O., Blondel M., Prettenhofer P., Weiss R., Dubourg V. (2011). Scikit-learn: Machine
learning in Python. J. Machine Learn. Res..

[39] Virtanen P., Gommers R., Oliphant T. E., Haberland M., Reddy T., Cournapeau D., Burovski E., Peterson P., Weckesser W., Bright J., van der Walt S. J., Brett M., Wilson J., Millman K. J., Mayorov N., Nelson A. R. J., Jones E., Kern R., Larson E., Carey C. J., Polat İ., Feng Y., Moore E. W., VanderPlas J., Laxalde D., Perktold J., Cimrman R., Henriksen I., Quintero E. A., Harris C. R., Archibald A. M., Ribeiro A. H., Pedregosa F., van Mulbregt P. (2020). SciPy 1.0
Contributors, SciPy 1.0: Fundamental Algorithms for Scientific Computing
in Python. Nat. Methods.

[40] Kabsch W. (1976). A solution
for the best rotation to relate two sets of vectors. Acta Crystallogr., Sect. A.

[41] Kabsch W. (1978). A discussion
of the solution for the best rotation to relate two sets of vectors. Acta Crystallogr., Sect. A.

[42] Crouse D. F. (2016). On implementing
2D rectangular assignment algorithms. IEEE Transactions
on Aerospace and Electronic Systems.

[43] Christensen A. S., Bratholm L. A., Faber F. A., Anatole von Lilienfeld O. (2020). FCHL revisited:
Faster and more accurate quantum machine learning. J. Chem. Phys..

[44] O’Boyle N. M., Banck M., James C. A., Morley C., Vandermeersch T., Hutchison G. R. (2011). Open Babel:
An open chemical toolbox. J. Cheminf..

[45] Müllner, D. Modern hierarchical, agglomerative clustering algorithms, 2011. https://arxiv.org/abs/1109.2378.

[46] Rousseeuw P. J. (1987). Silhouettes:
A graphical aid to the interpretation and validation of cluster analysis. Journal of Computational and Applied Mathematics.

[47] Caliński T., Harabasz J. (1974). A dendrite method for
cluster analysis. Commun. Statist..

[48] Davies D. L., Bouldin D. W. (1979). A Cluster Separation Measure. IEEE Transactions on Pattern Analysis and Machine Intelligence.

[49] Sokal R. R., James Rohlf F. (1962). The Comparison
of Dendrograms by Objective Methods. Taxon.

[50] Bussi G., Donadio D., Parrinello M. (2007). Canonical sampling through velocity
rescaling. J. Chem. Phys..

[51] Jorgensen W. L., Tirado-Rives J. (1988). The OPLS [optimized potentials for liquid simulations]
potential functions for proteins, energy minimizations for crystals
of cyclic peptides and crambin. J. Am. Chem.
Soc..

[52] Jorgensen W. L., Maxwell D. S., Tirado-Rives J. (1996). Development and Testing of the OPLS
All-Atom Force Field on Conformational Energetics and Properties of
Organic Liquids. J. Am. Chem. Soc..

[53] Jorgensen W. L., Chandrasekhar J., Madura J. D., Impey R. W., Klein M. L. (1983). Comparison
of simple potential functions for simulating liquid water. J. Chem. Phys..

[54] Gowers, R. J. ; Linke, M. ; Barnoud, J. ; Reddy, T. J. E. ; Melo, M. N. ; Seyler, S. L. ; Domański, J. ; Dotson, D. L. ; Buchoux, S. ; Kenney, I. M. ; Beckstein, O. MDAnalysis: A Python Package for the Rapid Analysis of Molecular Dynamics Simulations. Proceedings of the 15th Python in Science Conference, 2016; pp 98–105.

[55] Cezar H. M., Canuto S., Coutinho K. (2020). DICE: A Monte Carlo Code for Molecular
Simulation Including the Configurational Bias Monte Carlo Method. J. Chem. Inf. Model..

[56] Cezar H. M., Canuto S., Coutinho K. (2020). Understanding the absorption
spectrum
of mesityl oxide dye in solvents of different polarities. J. Mol. Liq..

[57] Lawrence J., Bernal J., Witzgall C. (2019). A Purely Algebraic
Justification
of the Kabsch-Umeyama Algorithm. J. Res. National
Inst. Stand. Technol..

[58] Sedgewick R. (1978). Implementing
Quicksort programs. Communications of the ACM.

[59] Tomizawa N. (1971). On some techniques
useful for solution of transportation network problems. Networks.

[60] Edmonds J., Karp R. M. (1972). Theoretical Improvements in Algorithmic Efficiency
for Network Flow Problems. Journal of the ACM.

