# Supplementary material for: clusttraj: A Solvent-Informed Clustering Tool for Molecular Modeling
Source: J Chem Theory Comput. 2025 Jul 3;21(14):6759–68. doi: 10.1021/acs.jctc.5c00634 (PMC12288011; doi:10.1021/acs.jctc.5c00634)
Supplement: Supplementary file 1 [file ct5c00634_si_001.pdf]

Supporting Information

# clusttraj: A Solvent-Informed Clustering Tool for Molecular Modeling

Rafael Bicudo Ribeiro[1], Henrique Musseli Cezar[2*]

[1] *Institute of Physics, University of São Paulo, Rua do Matão 1731, 05508-090 São Paulo, São Paulo, Brazil*

[2] *Hylleraas Centre for Quantum Molecular Sciences and Department of Chemistry, University of Oslo, PO Box 1033 Blindern, 0315 Oslo, Norway*

*✉: h.m.cezar@kjemi.uio.no

# Contents

**S1 Lysine solvated in water** **3**

Figure S1 - Medoid configurations from clusters (a) 1 and (b) 2 obtained with the Root Mean Square Deviation (RMSD) threshold set to maximize the SS. . . . . . . . . . . . . . . . . . . . . . . . . . . . . . 3

Figure S2 - KDE plot of the hydrogen bonds for each cluster with a threshold set to (a) optimize the SS and (b) to 5.5 Å. . . . . . . . . . . . . . 4

**S2 MOx solvated in methanol** **5**

Figure S3 - Superposition of MOx configurations for clusters (a) 1, (b) 2 and (c) 3. Methanol molecules are shown as cyan lines for better visualization. . . . . . . . . . . . . . . . . . . . . . . . . . . . . . . 5

Figure S4 - Dendrograms from the clustering of MOx and 4 methanol molecules using (a) single, (b) complete, (c) average, (d) weighted, (e) centroid, (f) median and (g) ward linkage methods. . . . . . . . . . . 6

**S3 Reliability of reordering algorithms** **7**

Table S1 - Clustering of randomly shuffled configurations generated from two unique configurations. The performance of each method is compared to trajectory files with 20, 100 and 500 configurations. . . . 7

**S4 Illustrative comparison with standard clustering approach** **9**

Table S2 - Clustering of randomly shuffled configurations generated from two unique configurations with `clusttraj` and TTClust packages. The performance of each method is compared to trajectory files with 20, 100 and 500 configurations. . . . . . . . . . . . . . . . . . . . . . 9

**S5 References** **11**

# S1 Lysine solvated in water

To investigate the effect of clustering into solvent-related properties, we considered a subset of the lysozyme protein comprising the lysine amino acid and the 10 closest water molecules to it. Since lysine has two oxygen and two nitrogen atoms, as shown in Figure S1, we computed the number of hydrogen bonds between the amino acid and water molecules for the configurations in each cluster.

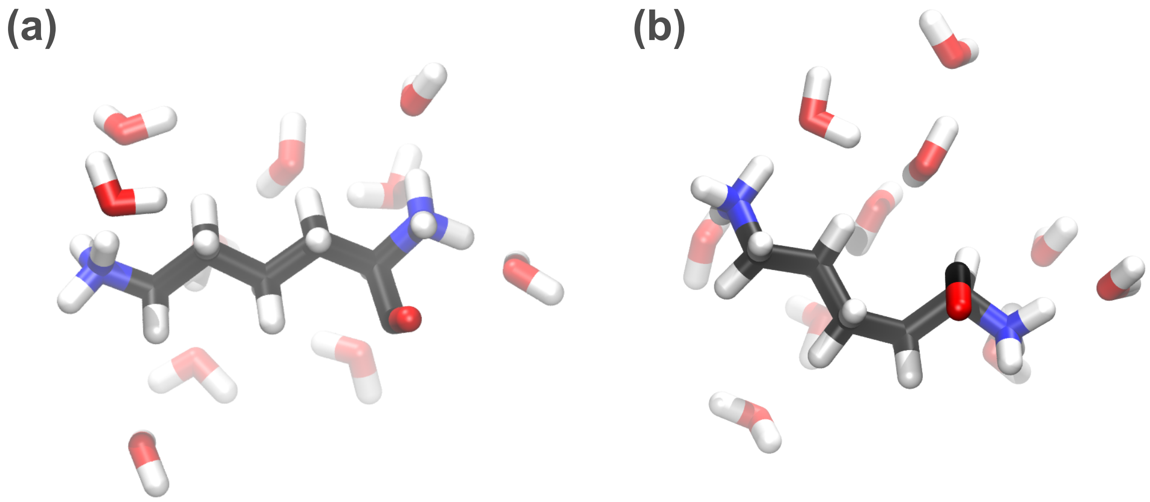


Figure S1: Medoid configurations from clusters (a) 1 and (b) 2 obtained with the Root Mean Square Deviation (RMSD) threshold set to maximize the SS.

The Hungarian and the Ward variance minimization methods were employed for the reordering and linkage scheme, respectively, and the total solute weight ($W^{\mathrm{solute}}$) was set to 0.5. The RMSD threshold was chosen to maximize the silhouette score (SS) [S6] and slightly decreased to 5.5 Å to increase the number of clusters from 2 to 3. Figure S2 presents the Kernel Density Estimation (KDE) plots [S4, S5] of the number of hydrogen bonds for each cluster obtained using both thresholds.

To some extent, the clustering procedure captured differences in the number of hydrogen bonds. As shown in Figure S2 (a), most configurations in Cluster 2 have less than 10 hydrogen bonds, while Cluster 1 has a more even distribution, achieving up to 30 H bonds. One extra cluster is formed when decreasing the threshold through the branching of Cluster 2, as shown in Figure S2 (b), and it changes the populations from 66 and 34 for clusters 1 and 2 to 66, 9 and 25 for clusters 1, 2 and 3, respectively. Despite the number of

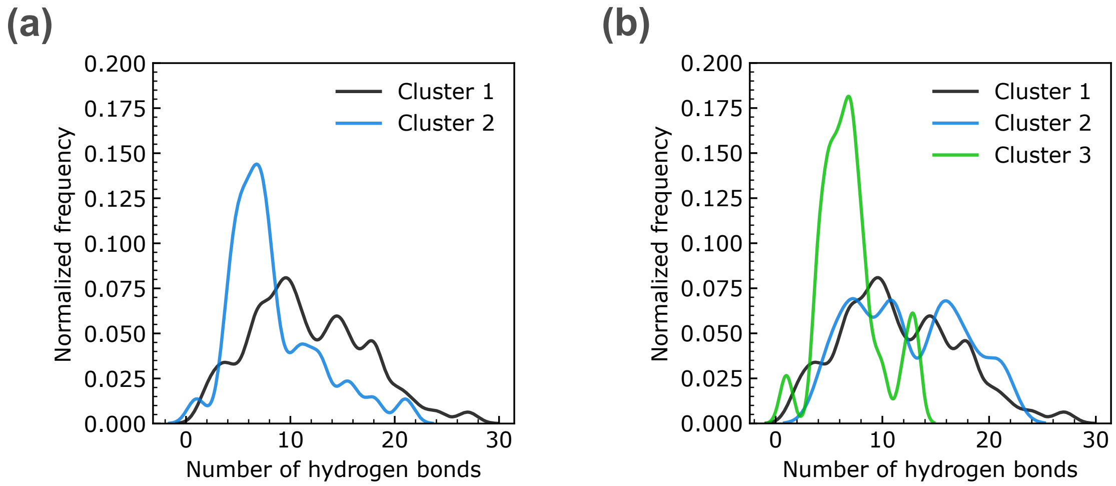


Figure S2: KDE plot of the hydrogen bonds for each cluster with a threshold set to (a) optimize the SS and (b) to 5.5 Å.

hydrogen bonds in configurations from Cluster 3 becoming even more localized, the newly formed Cluster 2 is similar to Cluster 1, reducing the heterogeneity between clusters.

Further refinement via solute weight tuning and considering different linkage schemes may improve the results. However, since establishing a hydrogen bond satisfies not only a distance but also an angular criterion [S1], the clustering via RMSD is likely to separate according to the distance but struggles to capture the subtle changes in the angle, especially for larger systems.

# S2 MOx solvated in methanol

As mentioned in Section 3.3.1, we performed a clustering procedure for mesityl oxide (MOx) and the four closest methanol molecules with an RMSD threshold of 10 Å. Considering the same weight for solute and solvent atoms, the Hungarian algorithm was combined with the Ward variance minimization method for the reordering and linkage schemes, respectively. As a result, we obtained three clusters and the superposition of configurations from each cluster is shown in Figure S3.

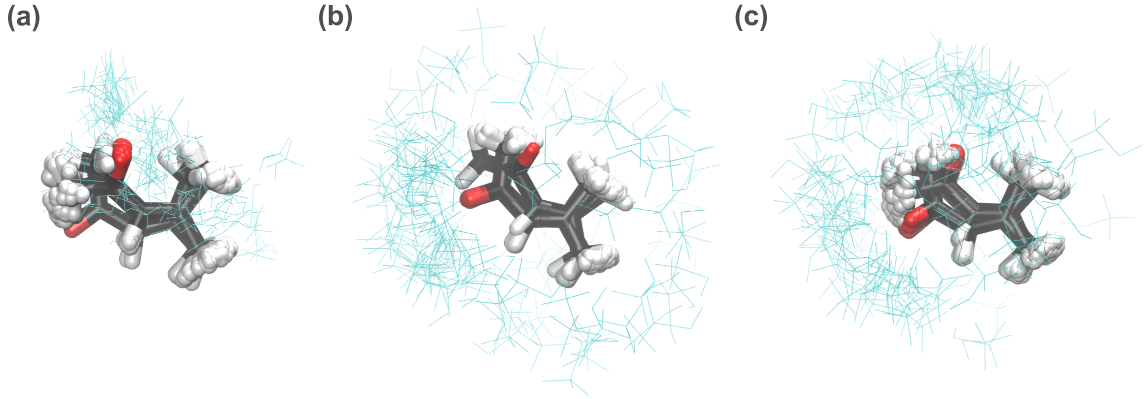


Figure S3: Superposition of MOx configurations for clusters (a) 1, (b) 2 and (c) 3. Methanol molecules are shown as cyan lines for better visualization.

In Section 3.3.1, we also compare different linkage schemes and the corresponding dendrograms are presented in Figure S4. The visual analysis shows that single, centroid, and median methods fail to form different clusters while increasing the similarity of configurations from the same cluster. This trend is in agreement with the goal of maximizing the CH score. As shown in Table 2, single, centroid and median have a CH score of 1.191, 3.186 and 3.043, respectively. On the other hand, average and Ward, the best-performing methods, yielded a Calinski Harabasz (CH) score [S2] of 6.285 and 10.110.

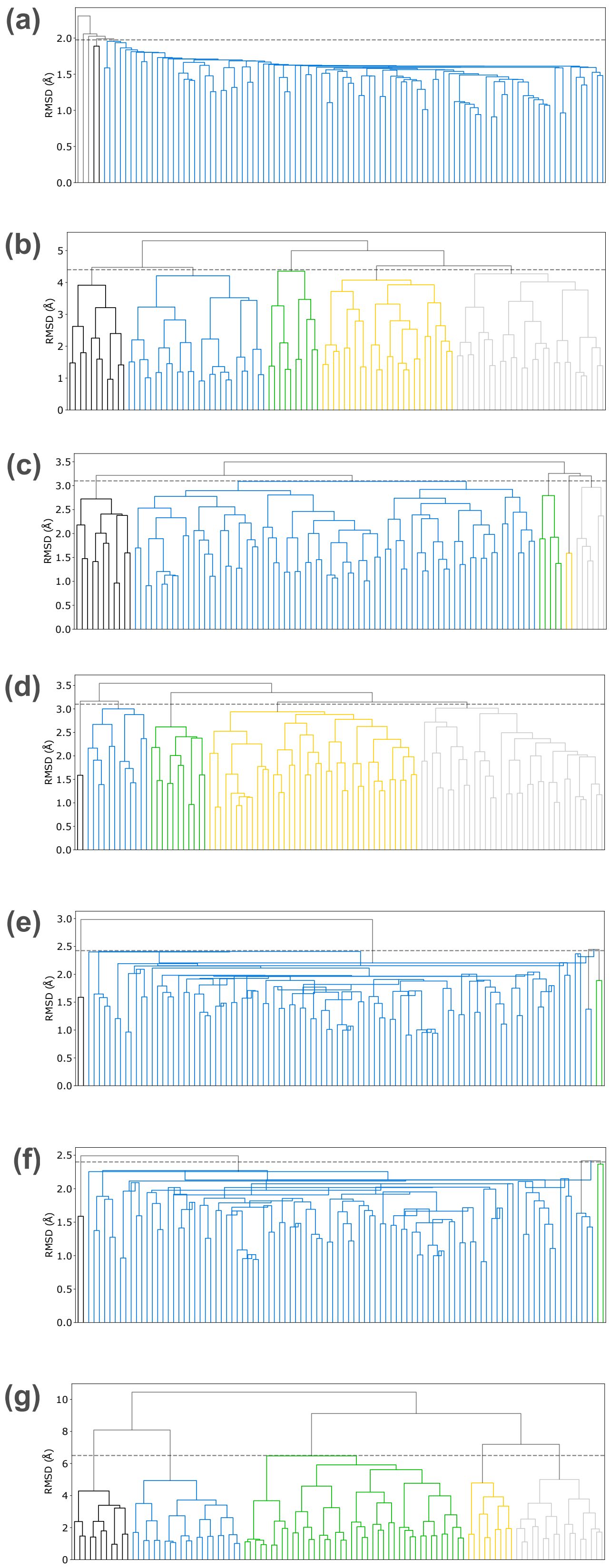


Figure S4: Dendrograms from the clustering of MOx and 4 methanol molecules using (a) single, (b) complete, (c) average, (d) weighted, (e) centroid, (f) median and (g) ward linkage methods.

# S3 Reliability of reordering algorithms

To compare the quality of the different reordering algorithms, we selected two representative conformations of MOx with four acetonitrile molecules. We used the brute force algorithm to reorder the atoms of the solvent molecules and found the optimal solution that minimizes the RMSD to 2.291 Å, using the implementation in the `rmsd` package [S3]. For both configurations, we randomly shuffled the indices of solvent atoms and further scrambled all "new" configurations into the same trajectory file. Then, we gradually increased the number of identical but shuffled configurations in the trajectory file, and each relabeling algorithm was employed. The number of clusters was set to maximize the silhouette coefficient and a final Kabsch rotation was performed. The results are summarized in Table S1.

Table S1: Clustering of randomly shuffled configurations generated from two unique configurations. The performance of each method is compared to trajectory files with 20, 100 and 500 configurations.

| Algorithm | Number of configurations | Number of clusters | Cluster index : size | RMSD (Å) : count |
|---|---|---|---|---|
| Hungarian | 20 | 2 | 1 : 10<br>2 : 10 | 0.000 : 100<br>2.314 : 100 |
| | 100 | 2 | 1 : 50<br>2 : 50 | 0.000 : 2500<br>2.314 : 2500 |
| | 500 | 2 | 1 : 250<br>2 : 250 | 0.000 : 62500<br>2.314 : 62500 |
| Distance | 20 | 2 | 1 : 10<br>2 : 10 | 0.000 : 100<br>4.335 : 100 |
| | 100 | 2 | 1 : 50<br>2 : 50 | 0.000 : 2500<br>4.335 : 2500 |
| | 500 | 2 | 1 : 250<br>2 : 250 | 0.000 : 62500<br>4.355 : 62500 |
| QML | 20 | 2 | 1 : 10<br>2 : 10 | 0.000 : 100<br>4.579 : 100 |
| | 100 | 2 | 1 : 50<br>2 : 50 | 0.000 : 5000<br>4.579 : 2500 |
| | 500 | 2 | 1 : 250<br>2 : 250 | 0.000 : 62500<br>4.579 : 62500 |

Despite being heuristic, the algorithms do not show any dependence on the trajec-

tory size, always converging to the same result. The correct number of clusters and the corresponding populations were consistently identified, as the minimized RMSD always converged to the same value, regardless of the atomic indices. In the last column of Table S1, we present the unique pairwise RMSD values between two snapshots and the number of times they appear in the RMSD matrix (count). Given the symmetry of the distance matrix, we only consider the values of the upper triangular part of the RMSD matrix.

As desired, only two values were reported, corresponding to the null RMSD between identical configurations and the non-zero RMSD between different configurations. Since the number of shuffled copies of each unique configuration is the same, one should expect equal proportions, as observed.

Comparing the non-zero RMSDs with the reference value allows us to assess the quality of each procedure. The Hungarian algorithm achieved the best results with a slight error of 0.023 Å, which should have a minor impact on the clustering procedure. On the other hand, both the distance and QML algorithms overestimated the RMSD by 2.044 Å and 2.288 Å, respectively. This trend between algorithms was also observed for the examples presented in the main text (see Section 3.4), but when considering a larger set of unique configurations, we found that QML tends to outperform the distance algorithm. For example, when considering the trajectories with 1 + 10 and 1 + 20 water molecules presented in Section 3.4, the sum of the RMSD matrix elements is 21 563 Å and 50 875 Å when using the distance algorithm but 21 157 Å and 27 962 Å with the QML algorithm, respectively. Nevertheless, the quality of heuristic algorithms can be system-dependent and should be carefully investigated for different applications.

## S4 Illustrative comparison with standard clustering approach

Considering the same trajectories used in Section S3, we performed an hierarchical clustering of the configurations using the well-established TTClust [S7] package. Since the trajectories are formed by shuffling the atomic indices of two unique configurations, we set the number of clusters as 2 when running TTClust. The results are shown in Table S2 and compared with `clusttraj` using the Hungarian algorithm.

Table S2: Clustering of randomly shuffled configurations generated from two unique configurations with `clusttraj` and TTClust packages. The performance of each method is compared to trajectory files with 20, 100 and 500 configurations.

| Program | Number of configurations | Number of clusters | Cluster index : size | Total time (s) |
|---|---|---|---|---|
| `clusttraj` | 20 | 2 | 1 : 10<br>2 : 10 | 5.23 |
| | 100 | 2 | 1 : 50<br>2 : 50 | 8.37 |
| | 500 | 2 | 1 : 250<br>2 : 250 | 36.46 |
| TTClust | 20 | 2 | 1 : 12<br>2 : 8 | 3.22 |
| | 100 | 2 | 1 : 68<br>2 : 32 | 3.28 |
| | 500 | 2 | 1 : 361<br>2 : 139 | 4.06 |

As anticipated, even though we predefined the correct number of clusters, the lack of a reordering scheme resulted in non-physical clusters with varying populations, depending on the trajectory size. Since the reordering scheme is the most computationally demanding step, scaling with $\mathcal{O}(N^3)$ for the Hungarian algorithm, where $N$ is the number of atoms from identical molecules (see Section 3.4 of the main text), the overall run time with TTClust is shorter. However, despite the computational cost of `clusttraj`'s approach being higher, the wall time is only a few seconds even for the larger trajectory with 500 configurations. Considering that these calculations were performed on a commercial

laptop, one should be able to benefit from the parallel implementation of `clusttraj` and cluster configurations from larger trajectories (with more atoms and/or more snapshots) using dedicated high-performance computing resources.

## S5 References


[S1] Elangannan Arunan, Gautam R. Desiraju, Roger A. Klein, Joanna Sadlej, Steve Scheiner, Ibon Alkorta, David C. Clary, Robert H. Crabtree, Joseph J. Dannenberg, Pavel Hobza, Henrik G. Kjaergaard, Anthony C. Legon, Benedetta Mennucci, and David J. Nesbitt. Definition of the hydrogen bond (IUPAC Recommendations 2011). *Pure and Applied Chemistry*, 83:1637–1641, 2011.

[S2] T. Caliński and J. Harabasz. A dendrite method for cluster analysis. *Communications in Statistics*, 3:1–27, 1974.

[S3] Jimmy C. Kromann. Calculate root-mean-square deviation (rmsd) of two molecules using rotation. `https://github.com/charnley/rmsd`, 2025.

[S4] Emanuel Parzen. On Estimation of a Probability Density Function and Mode. *The Annals of Mathematical Statistics*, 33:1065–1076, 1962.

[S5] Murray Rosenblatt. Remarks on Some Nonparametric Estimates of a Density Function. *The Annals of Mathematical Statistics*, 27:832–837, 1962.

[S6] Peter J. Rousseeuw. Silhouettes: A graphical aid to the interpretation and validation of cluster analysis. *Journal of Computational and Applied Mathematics*, 20:53–65, 1987.

[S7] Thibault Tubiana, Jean-Charles Carvaillo, Yves Boulard, and Stéphane Bressanelli. TTClust: A Versatile Molecular Simulation Trajectory ClusteringProgram with Graphical Summaries. *Journal of Chemical Information and Modeling*, 58:2178–2182, 2018.
